# Treatise on Dental Caries

**Published:** 1879-02

**Authors:** 


*Treatise on Dental Caries, and Experimental and Therapeutic Investigations.* By Dr. E. Magitot.

We simply announce the receipt of this most valuable and interesting original work, in this number of the JOURNAL, promising to give it in another place the attention it deserves. It would have been noticed before this, but for the illness of the writer. H.

